# The Intolerance of Uncertainty and “Untact” Buying Behavior: The Mediating Role of the Perceived Risk of COVID-19 Variants and Protection Motivation

**DOI:** 10.3389/fpsyg.2022.807331

**Published:** 2022-01-31

**Authors:** Shunying Zhao, Baojuan Ye, Weisha Wang, Yadi Zeng

**Affiliations:** ^1^School of Education Science, Jiaying University, Meizhou, China; ^2^School of Psychology, Jiangxi Normal University, Nanchang, China; ^3^Center of Mental Health Education and Research, School of Psychology, Jiangxi Normal University, Nanchang, China; ^4^Newcastle University Business School, Newcastle University, Newcastle upon Tyne, United Kingdom; ^5^Center of Preschool Education, Mental Health Education and Research, School of Psychology, Jiangxi Normal University, Nanchang, China

**Keywords:** intolerance of uncertainty, perceived risk of COVID-19 variants, protection motivation, untact, buying behavior

## Abstract

Draw on the protection motivation theory, this study investigated the impacts of intolerance of uncertainty on “untact” buying behavior, and examined the sequential mediating role of the perceived risk of COVID-19 variants and protection motivation. A total of 1,564 (*M*_*age*_ = 20.75, *SD* = 1.92) young individuals participated in the survey. The serial mediation analysis results reveal that intolerance of uncertainty influences one’s “untact” buying behavior through “perceived risk of COVID-19 variants - protection motivation.” Both internal (intolerance of uncertainty, protection motivation) and external (risk of COVID-19) factors worked together to accelerate the transition of individuals’ consumption behavior during COVID-19 pandemic. Therefore, our findings generate important implications for public mental health and economic recovery in the post-COVID-19 era.

## Introduction

“Untact” refers to emerging human behavior that minimizes the physical interaction, which is a new word first coined by South Korean scholars through adding the prefix “un” (i.e.,“no”) to the word “contact” (Lee et al., 2019; Kim and Sung, 2020). Similarly, “untact service” is a consumption service without physical interactions between staff and consumers via the use of digital technologies such as computers and mobile phones (Jmour, 2020; Lee and Lee, 2020). The “untact” buying behavior is generally launched by individuals who intend to consume services without physical interactions (Fitzsimmons et al., 2008). Studies in recent years have shown that, due to the wide application of information advanced technology in commercial activities, the “untact” buying behavior of the public has already shown an increasing trend before the COVID-19 pandemic outbreak (Lee et al., 2019; Lee and Lee, 2020).

After the COVID-19 outbreak, a sharp increase in “untact” buying behavior is observed all around the world. For example, Li et al. (2020) claim that in comparison with the time before COVID-19, 38% farmers market consumers and 34% supermarket shoppers moved to buy items online during the pandemic outbreak. During COVID-19, the value of online sales of South Korea amounted to more than 159 trillion South Korean won, which experienced an unexpected increase amidst the ongoing downward trend since 2017 (Statista Research Department, 2021). In the United States, Amazon’s online sales sharply surged during the COVID-19 pandemic, which ranked first in the e-commerce sector of the United States with a market share of 38% (Liu, 2020).

According to CNBC, young Chinese consumers (18–30 years old) accounts for 17% of the whole population but contribute to 25% of total expenditure on new brands (Bala, 2021). More importantly, the spending power of Chinese consumers will double in 10 years and reach $12.7 trillion by 2030 (Bala, 2021). To what extent that COVID-19 may accelerate the transition of individuals’ buying behavior from contact buying to “untact” buying remain unknown. Exploring antecedent factors of “untact” buying behavior during the COVID-19 pandemic is not only valuable for revealing the process mechanism of the changing buying behavior trend in a public health crisis context, but also helpful to reveal its survival significance of human beings. After the COVID-19 outbreak, there are several studies try to explain the reasons why “untact” buying behavior increases explosively, but those studies mainly focused on the characteristics of the COVID-19 itself, such as social distance for preventing the spread out of COVID-19 and the public’s fear of virus infection risk (Ali, 2020; Khan, 2021).

The organism-environment interaction model (Lerner et al., 2006) proposes that human behavior is shaped by the dynamic interactions between external environmental variables and internal personality variables. However, to the best of our knowledge, there is no research exploring the associations between internal personality variables and “untact” buying behavior during the COVID-19 pandemic. Therefore, we aim to fill in this gap by taking the effects of both internal personality difference variables (e.g., intolerance of uncertainty, protection motivation) and environmental-external factor (e.g., perceived risk of COVID-19 variants) into consideration together and building a chain mediation model. This research, therefore, not only sheds light on the psychological mechanism that underlie one’s buying behavior transition during the COVID-19 pandemic but also emphasizes the survival implications of “untact” buying behavior, which is essential for global economic growth and generate jobs.

## Literature Review and Hypotheses Development

### Intolerance of Uncertainty and “Untact” Buying Behavior

The state of uncertainty refers to the doubt that exists about whether a particular negative outcome will occur (Keren and Gerritsen, 1999), the doubt is a powerful stressor with both psychological and physiological consequences for individuals (Rosen et al., 2014). Intolerance of uncertainty refers to individuals’ cognitive, emotional, and behavioral reactions to uncertainty states (Freeston et al., 1994). Besides, intolerance of uncertainty is a trait characteristic that arises from negative beliefs about uncertainty and its consequences (Rosen et al., 2014), people with a high intolerance of uncertainty can’t bear the aversive response triggered by perception of uncertain situation (Carleton, 2016). Therefore, intolerance of uncertainty is a critical internal personality difference variable that inevitably affects individuals’ “untact” buying behavior during COVID-19 pandemic.

On the one side, one of the main motivators for “untact” buying behavior is to eliminate potential uncertain factors during the purchase process. For example, such uncertain factors include uncomfortable interactions with employees, personal information leakage, and to increase their control over business transactions (Kim and Sung, 2020). On the other side, with the ultimate goal to eliminate uncertainties, individuals with a high level of intolerance of uncertainty have excessive planning and preparation behavioral intention for negative risks or threats (Brouwers and Sorrentino, 1993). Recently, some investigations find that individuals’ intolerance of uncertainty is positively associated with avoidance behavior to protect themselves (Di Giuseppe and Taylor, 2021; Durak Batıgün and Şenkal Ertürk, 2021). To protect themselves from threats arises from uncertainty and avoid negative feelings during the contact buying process, individuals with a high intolerance of uncertainty may tend to adopt “untact” buying behavior. Thus, based on the reasoning and literature described above, the following hypothesis is proposed:

H1: The intolerance of uncertainty positively influences one’s “untact” buying behavior. In other words, individuals who score higher (vs. lower) on intolerance of uncertainty are expected to have a higher (vs. lower) intention to engage in “untact” buying behavior during the COVID-19 pandemic.

### The Mediating Role of the Perceived Risk of COVID-19 Variants

Risk perception refers to individuals’ psychological evaluations of the probability and consequences of an adverse outcome (Sjöberg, 2000). Research indicates that intolerance of uncertainty was positively link to individuals’ risk perception (Dugas et al., 2005; Koerner and Dugas, 2008; Taha et al., 2014). Intolerance of uncertainty act as a cognitive filter through which the uncertainty situation is perceived as unacceptable and discomfort (Buhr and Dugas, 2002), which makes it an important negative cognitive factor for risk perception (Koerner and Dugas, 2008). People with a higher intolerance of uncertainty tend to have a higher level of risk perception due to their cognitive interpretation bias, which leads to individuals interpret neutral information as threatening (Dugas et al., 2005). Furthermore, Taha et al. (2014) claim that individuals with a higher level of intolerant of uncertainty are more likely to have a higher level of H1N1- related anxiety and perceive the H1N1 pandemic as a threatening factor. Recently, the positive association between intolerance of uncertainty and risk perception of the COVID-19 pandemic was already supported by ample empirical researches (Asmundson and Taylor, 2020; Bakioğlu et al., 2020; Mertens et al., 2020; Satici et al., 2020; Tull et al., 2020).

There is also some evidence that individuals’ risk perception of disease can arouse their protective behavior. As individuals’ subjective judgment of risky situations or events (Slovic, 1987), risk perception tends to play a critical role in influencing their protective behaviors during the COVID-19 pandemic (Dai et al., 2020). According to Floyd et al. (2000), the ultimate goal of human behavior is to avoid or reduce risk factors they perceived. Given the fast spread of the COVID-19 virus, people often perceive “untact” buying behavior as a self-protective behavior to avoid virus infection risk (Bae and Chang, 2020). In a similar vein, Perić et al. (2021) suggest that tourists’ health and psychological risk perception of COVID-19 may negatively predict their travel intentions. Earlier research during the pandemic outbreak (Cowling et al., 2010; Ibuka et al., 2010; van der Weerd et al., 2011; Tooher et al., 2013; Bali et al., 2016), and the current COVID-19 pandemic (Al-Rasheed, 2020; Kaspar, 2020; Shahnazi et al., 2020; Jose et al., 2021) find that risk perception is positively related to protective behavior during pandemics. It has also been found that intolerance of uncertainty may predict more approach-oriented coping behavior when faced with an uncertain health threat (Rosen and Knäuper, 2009).

According to the Health Belief Model, perceived health risks are the key factors that encourage individuals’ health-promoting behaviors (Champion and Skinner, 2008). In general, individuals tend to exhibit protective behaviors when they face serious threats and often perceive themselves as members of vulnerable groups (Milne et al., 2000). During the COVID-19 outbreak, the “untact” buying behavior may be perceived as health-promoting behavior and protecting individuals from the COVID-19 variants. Similarly, Yastibaş and Akpinar (2021) propose that individuals’ illness perceptions can mediate the relationshi between personality traits and health-related behavior. Therefore, it is reasonable to expect that the perceived risk of COVID-19 variants is positively related to “untact” buying behavior. Thus, based on the reasoning and literature described above, we propose that:

H2: The perceived risk of COVID-19 variants mediates the relationship between in tolerance of uncertainty and “untact” buying behavior.

### The Mediating Role of Protection Motivation

Protection Motivation Theory (PMT, Rogers, 1983), a comprehensive psychological model, has been widely used in predicting protective behaviors in epidemic outbreak backgrounds such as H1N1 and COVID-19 (Milne et al., 2000; Bish et al., 2011; Sharifirad et al., 2014; Al-Rasheed, 2020). PMT proposes that individuals use certain cognitive belief patterns to protect themselves from danger in uncertain contexts (Schmees, 2020). The integrative uncertainty tolerance model suggests that perception of the uncertainty of external stimuli could trigger a variety of cognitive and emotional reactions (Hillen et al., 2017), which in turn affect the motivation factors.

Intolerance of uncertainty is an anxiety-related reaction (Greco and Roger, 2001; Laugesen et al., 2003), and anxiety-related reaction can lead to strong protective motivation. High intolerance of uncertainty may lead to negative feelings such as anxiety and worry under COVID-19, which in turn may lead to protective motivation to wipe away these negative feelings. For this reason, intolerance of uncertainty, as an especial cognitive belief pattern to uncertainty (Freeston et al., 1994), is an important antecedent of protective motivation under the COVID-19 outbreak. Besides, the protective motivations of “untact” buying behavior have been studied before the COVID-19 outbreak. For example, Kim and Sung (2020) claim that three protective motivations may lead to “untact” buying behavior of individuals: (1) to protect their personal information, (2) to increase their control over business transactions, and (3) to avoid uncomfortable interactions with employees.

In the public health context, researchers often argue that protection motivation explains why some individuals protect themselves from a health threat event or situation (Lwin et al., 2010). Therefore, as protective behavior under COVID-19, “untact” buying behavior is the outcome of protective motivation due to risk perception of COVID-19 variants infection. Akter et al. (2021) also argue that the health and safety concern tend to cause a remarkable change in one’s buying behavior, such as in-home delivery of goods, which is breaking the old habits of physically going to brick and mortar places. Thus, we proposed the mediating effect hypothesis:

H3: Protection motivation mediates the relationship between intolerance of uncertainty and “untact” buying behavior.

### Perceived Risk of COVID-19 Variants and Protection Motivation

It is well established that protection motivation is affected by the perceived risk of external environmental factors and mediates the relationship between perceived risk and protective behavior (Rogers, 1975; Champion and Skinner, 2008; Bae and Chang, 2020). In the early times, Kagan (1972) suggests that risk reduction was a direct factor affecting protection motivation when people are in dangerous. Rogers (1975) also insists that people’s perceived risk evokes their protection motivation, which in turn leads to protective behaviors. In addition, the health belief model, a widely used conceptual framework explaining individuals’ health behaviors, also suggests that individuals who perceive a high level of risk to their health tend to have stronger protection motivation, which in turn increase their engagement in health-protective behaviors to cope with their risk perception and negative emotions (Rosenstock, 1974).

After the outbreak of COVID-19, several investigations have already supported the positive association between perceived risk of COVID-19 variants and protection motivation. For example, Bae and Chang (2020) point out that compared with the previous pandemic such as SARS or MERS, the COVID-19 pandemic is far more infectious, which activates higher protection motivation. Similarly, in the field of tourism research, Zheng et al. (2021) find that threat severity and susceptibility of COVID-19 leads to protection motivation and follow-up protective behaviors. The risk of the COVID-19 pandemic can trigger protection motivation of the public (Kim and Sung, 2020), which makes the “untact” buying behavior visible across all contexts within the society. Aforementioned, it is reasonable to believe that:

H4: The effects of intolerance of uncertainty on “untact” buying behavior will be mediated by the path of perceived risk of COVID-19 variants-protection motivation.

### The Present Study

**FIGURE 1:**
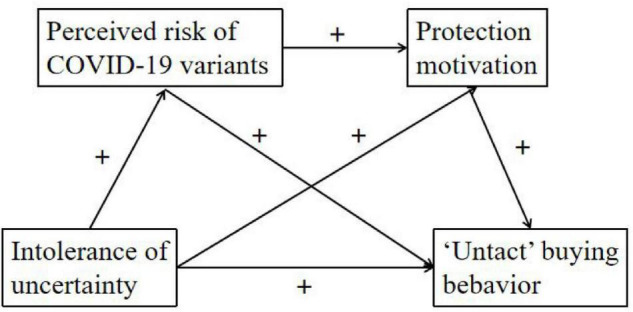
The conceptual model. + means positive relationship.

Taken together, the current study develops the serial mediation conceptual model (Figure 1). We first examined whether intolerance of uncertainty is positively associated with “untact” buying behavior. Secondly, the study examined whether perceived risk of COVID-19 variants mediate the link between intolerance of uncertainty and “untact” buying behavior. Thirdly, the study examined whether protection motivation mediate the link between intolerance of uncertainty and “untact” buying behavior. Lastly, the study tested the hypothesis that intolerance of uncertainty relates to “untact” buying behavior through the path of perceived risk of COVID-19 variants to protection motivation.

## Materials and Methods

### Participants and Procedures

#### Participants

A total of 1,636 participants completed this investigation. After eliminating ineligible observations (e.g., participants completed the survey within 60 s), 1564 participants with an effective response rate of 95.60% were included in the full analyses. Among the 1564 participants, ranging in age from 18 to 30 years old (*M* = 20.75, *SD* = 1.92; 66.6% female). Among these participants, 5.6% less than 1 year of online buying experience, 38.1% between 1 and 3 years, 41.3% between 4 and 6 years, 15% over 6 years. All participants volunteered to participate in this investigation and could take part in a lottery for 1–3 RMB as compensation.

#### Procedures

This investigation was conducted in the period from 4 February 2021 to 9 February 2021, at which time a large-scale of COVID-19 variants broke out in China. To abide by the epidemic prevention policy and reduce face-to-face contact, the method of this investigation’s data collection was convenience sampling. We distributed the questionnaire to potential participants electronically via SurveyStar (Chinese online data collection software; Changsha Ranxing Science and Technology, Shanghai, China) and distributed on WeChat and (Chinese social media platform). Participants can’t submit the questionnaire successfully online until all items were fully completed. All participants were informed of the purpose and anonymity of this survey prior to their participation.

### Measures

#### Intolerance of Uncertainty

The Intolerance of Uncertainty Scale-12 (IUS-12) was originally revised by Carleton et al. (2007). To adapt to the Chinese context, this study used the Chinese version of IUS-12 (Wu et al., 2016). The IUS-12 has 12 items including 2 dimensions: (a) 7 items of prospective anxiety (e.g., “Unforeseen events upset me greatly”), (b) 5 items of inhibitory anxiety (e.g., “Uncertainty keeps me from living a full life”). All items were measured on a five-point Likert scale (1 = totally disagree to 5 = totally agree). Higher scores indicated greater intolerance of uncertainty. In the current study, The Cronbach’s α for scale scores was 0.909. CFA showed acceptable fit for the revised scale, CFI = 0.960, TLI = 0.943, SRMR = 0.073, and RMSEA = 0.040.

#### “Untact” Buying Behavior

To measure individuals’ “untact” buying behavior 5 items from previous studies was revised (Li and Ma, 2018; Zheng et al., 2021). The newly revised scale has two factors: (a) 3 items of online buying preference (e.g., “Online buying is my first choice during COVID-19 outbreak period”), (b) 2 items of buying offline avoidance (e.g., “I try to avoid buying in brick-and-mortar stores during COVID-19 period”). All items were measured on a five-point Likert scale (1 = totally disagree to 5 = totally agree). Higher score indicates more online buying behavior. The Cronbach’s α of the current study is 0.859. CFA shows acceptable fit for the revised scale, CFI = 0.999, TLI = 0.996, RMSEA = 0.033, and SRMR = 0.006.

#### Perceived Risk of COVID-19 Variants

To measure one’s perceived risk of COVID-19 variants, this study revised the COVID-19 Perceived Risk Scale (CPRS) (Yıldırım and Güler, 2020), which was adapted from SARS Risk Perception Scale (Brug et al., 2004). To adapt the COVID-19 variants, this study mainly revised the expression such as replacing “COVID-19” with “COVID-19 variants” and deleted the item “perceived likelihood of other diseases (e.g., diabetes/asthma).” The newly revised scale measured two dimensions: (a) 3 items of cognitive dimension (e.g., “I am more likely to be infected with COVID-19 variants compared to other persons”), (b) 4 items of emotional dimension (e.g., “I am worried that I will be infected with COVID-19 variants”). Each item is rated on a Likert scale ranging from 1 (totally disagree) to 5 (totally agree). Higher score reflects higher levels of risk related to COVID-19 variants. The Cronbach’s α of the current scale is 0.722. CFA showed acceptable fit for the revised scale, CFI = 0.984, TLI = 0.962, SRMR = 0.083. RMSEA = 0.031.

#### Protection Motivation

To measure individuals’ motivation to engage in protective behavior under COVID-19, this study revised three items (e.g., “I must protect myself from being infected by COVID-19 when I buy something outside”) that have been widely used in protective behavior studies (Posey et al., 2015; Zheng et al., 2021). Each item is rated on a Likert-type scale ranging from 1 (strongly disagree) to 5 (strongly agree). Higher score indicates stronger protection motivation. In the present study, Cronbach’s α is 0.833. Furthermore, CFA results suggest that the one-factor model was a saturation model, which meant that the scale of protection motivation had good validity.

### Data Analysis

First, we used SPSS 20.0 to conduct descriptive statistics and correlation analysis between dependent variables and independent variables. Tests of normality revealed that the study variables showed no significant deviation from normality (i.e., Skewness < |2.0| and Kurtosis < |7.0|, see Table 1) (Curran et al., 1996). Then, we conducted a serial mediation analysis by PROCESS Models 6 macro for SPSS (Hayes, 2013) to examine the indirect effect of intolerance of uncertainty on “untact” buying behavior through the perceived risk of COVID-19 variants and protection motivation. Bootstrap confidence intervals (CIs) analysis was carried out to determine whether the effects in Model 6 were significant based on 5000 random samples (Hayes, 2017). The effect was significant if the CIs do not include zero. All main research variables were standardized prior to being analyzed.

**TABLE 1:** Descriptive analysis results.

Variables	Mean	*SD*	1	2	3	4
1. IUS	3.272	0.626	1			
2. PRCV	2.798	0.699	0.299***	1		
3. PM	4.088	0.723	0.159***	0.147***	1	
4. UBB	3.720	0.691	0.234***	0.223***	0.496***	1
5. Skewness	−	−	–0.512	–0.411	–1.256	–0.560
6. Kurtosis	−	−	1.140	–0.313	2.787	1.028

N = 1564. M, mean; SD, standard deviation; IUS, intolerance of uncertainty; PRCV, perceived risk of COVID-19 variants; PM, protection motivation; UBB, “Untact” buying behavior. ***p < 0.001.

## Results

### Correlation Analyses

The means, *SDs*, and Pearson correlations were shown in Table 1. After control the variables such as gender, years of study, and online shopping experience, the results indicated that intolerance of uncertainty was positively related with perceived risk of COVID-19 variants (*r* = 0.299, *p* < 0.001), protection motivation (*r* = 0.159, *p* < 0.001), and “untact” buying behavior (*r* = 0.234, *p* < 0.001). Thus, H1 is supported. Furthermore, perceived risk of COVID-19 variants was positively related to protection motivation (*r* = 0.147, *p* < 0.001) and “untact” buying behavior (*r* = 0.223, *p* < 0.001). Finally, protection motivation was positively correlated with “untact” buying behavior (*r* = 0.496, *p* < 0.001).

### The Serial Mediating Effects Analyses

We conducted the serial mediation analysis to examine the mediating role of perceived risk of COVID-19 variants and protection motivation. Table 2 shows the serial mediation analysis results. The mediation analysis results showed that the indirect effect of intolerance of uncertainty on “untact” buying behavior through perceived risk of COVID-19 variants [β = 0.035, *SE* = 0.007, 95% *CI* = (0.021, 0.050)] was significant but low, thus H2 was supported. The results showed a significant indirect effect through protection motivation [β = 0.058, *SE* = 0.017, 95% CI = (0.026, 0.091)], thus H3 is supported. Furthermore, the direct effect of intolerance of uncertainty on “untact” buying behavior [β = 0.126, *SE* = 0.023, 95% CI = (0.082, 0.170)] was also significant. Additionally, the serial mediation analysis results revealed that the path of intolerance of uncertainty→perceived risk of COVID-19 variants→protection motivation→ “untact” buying behavior was significant but low [β = 0.015, *SE* = 0.004, 95% *CI* = (0.008, 0.023)], thus H4 was supported. The overall regression analysis results show that intolerance of uncertainty, perceived risk of COVID-19 variants, and protection motivation explained 46.154% of the variance in “untact” buying behavior (see Table 3).

**TABLE 2:** Regression coefficients and standard errors for the serial mediation model presented in Figure 2.

	Consequent								
	PRCV			PM			UBB		
Antecedent	Coefficient	*SE*	*p*	Coefficient	*SE*	*p*	Coefficient	*SE*	*p*
IUS	0.297	0.024	< 0.001	0.125	0.026	< 0.001	0.126	0.023	< 0.001
PRCV				0.11	0.026	< 0.001	0.118	0.023	< 0.001
PM							0.460	0.022	< 0.001
Gender	0.013	0.051	>0.05	−0.152	0.053	< 0.01	−0.031	0.046	>0.05
YOS	−0.119	0.024	< 0.001	−0.056	0.025	< 0.05	0.016	0.022	>0.05
OSE	0.007	0.032	>0.05	−0.008	0.033	>0.05	0.069	0.029	< 0.05
	*R*^2^= 0.102			*R*^2^= 0.046			*R*^2^= 0.289		
	*F* (4,1559) = 44.101			*F* (5,1558) = 14.973			*F* (6,1557) = 105.264		
	*p* < 0.001			*p* < 0.001			*p* < 0.001		

*N = 1,564. IUS, intolerance of uncertainty; PRCV, perceived risk of COVID-19 variants; PM, protection motivation; UBB, “Untact” buying behavior; YOS, years of study; OSE, online shopping experience.*

**FIGURE 2:**
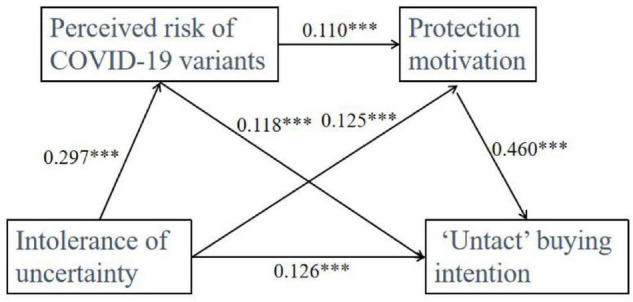
The serial mediation model. ***p < 0.001.

**TABLE 3:** Mediating effect test.

	Path way	Coefficient	Bootstrap 95% CI	Effect ratio (%)
Total effects		0.234	0.185–0.282	
Direct effects		0.126	0.082–0.170	53.846
Total indirect effects		0.108	0.071–0.146	46.154
Indirect effects	1.IUS-PRCV-UBB	0.035	0.021–0.050	32.407
	2.IUS-PM-UBB	0.058	0.026–0.091	53.704
	3.IUS-PRCV-PM-UBB	0.015	0.008–0.023	13.889

*N = 1,564. IUS, intolerance of uncertainty; PRCV, perceived risk of COVID-19 variants; PM, protection motivation; UBB, “untact” buying behavior.*

## Discussion

To our best knowledge, this study is the first one to focus on the relationships between individuals’ intolerance of uncertainty and “untact” buying behavior during the COVID-19 pandemic. The present study tested the relationship between intolerance of uncertainty and “untact” buying behavior among a large sample of Chinese university students during the COVID-19 period. The results suggest that one’s intolerance of uncertainty is positively related to “untact” buying behavior. The perceived risk of COVID-19 variants and protection motivation independently mediated the effects of intolerance of uncertainty on one’s “untact” buying behavior. Besides, the perceived risk of COVID-19 variants and protection motivation were found to play serial mediating roles in the relationship between intolerance of uncertainty and “untact” buying behavior. These findings generated important theoretical implications for understanding the relationships between psychological motivation, psychological needs, and behavior of individuals with different intolerance of uncertainty in crisis.

The present study sheds light on the complex nature of “untact” buying behavior development by examining whether and how intolerance of uncertainty was associated with the rising of “untact” buying behavior among Chinese University students. First, our data revealed that intolerance of uncertainty was an important factor to “untact” buying behavior during the COVID-19 pandemic. Second, in accordance with the organism-environment interaction model and protection motivation theory, our findings indicated that perceived risk of COVID-19 variants (threat assessment) and protection motivation (intensity of demand for security) may link intolerance of uncertainty (negative beliefs about risk factor) to “untact” buying behavior (protective behavior). Our results concurred with the serial mediator model, suggesting that high intolerance of uncertainty exacerbates individuals’ subsequent perceived risk of COVID-19 variants, enhancing their protection motivation, which increases “untact” buying behavior.

Firstly, our findings show that individuals with a higher level of intolerance of uncertainty tend to exhibit more “untact” buying behavior than those with a lower level of intolerance of uncertainty. Our results provide further support to Golets et al. (2021), who suggest that highly uncertainty-intolerant individuals tend to reveal protective behavior such as reschedule their trips during the COVID-19 pandemic. Individuals may demonstrate anxiety-related reactions to uncertain events or environments (Freeston et al., 1994), thus, individuals with a high level of intolerance of uncertainty tend to show the “untact” buying behavior to eliminate anxiety or worry due to uncertainty.

Secondly, our results illustrate that one’s perceived risk of COVID-19 variants partially mediates the relationship between the level of their intolerance of uncertainty and “untact” buying behavior. Consistent with prior research (Taha et al., 2014; Asmundson and Taylor, 2020; Bakioğlu et al., 2020; Tull et al., 2020), our findings provide further evidence to support the positive relationship between intolerance of uncertainty and perceived risk of COVID-19 variants Furthermore, our findings are also in line with prior research on protective behavior (Cowling et al., 2010; Bae and Chang, 2020; Kim and Sung, 2020; Perić et al., 2021), and revealed a positive relationship between the perceived risk of pandemics and individuals’ protective behavior. Individuals with a higher level of intolerance of uncertainty often have a negative cognitive interpretation bias (Dugas et al., 2005), which made them perceived more risk of COVID-19 variants. At the same time, based on HBM (Champion and Skinner, 2008), perceived high health risks of COVID-19 variants may trigger individuals’ protective behaviors.

Thirdly, we also found that protection motivation mediated the association between intolerance of uncertainty and “untact” buying behavior. First, we found that students’ intolerance of uncertainty has a positive relationship with their protection motivation. According to Kim and Sung (2020), individuals tend to reveal their “untact” buying behavior to eliminate the potential uncertainty factors which are caused by negative consequences in the transaction process. Individuals with a high level of intolerance of uncertainty tend to be more alert to uncertain factors in their daily life, generating protection motivation than their counterparts. Second, in line with the previous findings, individuals with a high level of protection motivation tend to involve in “untact” buying behavior (Bae and Chang, 2020; Akter et al., 2021). The possible explanation may be that individuals with a high level of intolerance of uncertainty experience more negative emotions such as worry and anxiety. While negative emotions can evoke protection motivation, which in turn arouse protective behavior (Rogers, 1975).

Lastly, our results indicated that intolerance of uncertainty would exert an influence on “untact” buying behavior among individuals through the serial mediation links of the perceived risk of COVID-19 variants and protection motivation during the COVID-19 pandemic. To date, many studies have found that individuals’ perceived risk of COVID-19 variants has a positive association with their protection motivation (Bae and Chang, 2020; Zheng et al., 2021). Besides, Kagan (1972) suggests that risk and negative emotional reduction are direct factors affecting individuals’ protection motivation under COVID-19. This research result can be explained by the organism-environment interaction model (Lerner et al., 2006), which points out individual’s behavior is the outcome of the dynamic interactions between internal factors and external environmental factors. Therefore, “untact” buying behavior can be influenced by both internal factors such as intolerance of uncertainty and protection motivation, as well as by external factors such as the risk of COVID-19 variants infection.

## Theoretical and Practical Implications

This study is the first one to explore the associations among intolerance of uncertainty, perceived risk of COVID-19 variants, “untact” buying behavior and protection motivation after COVID-19 variants emerged. Theoretically, this study generates insightful contributions to the literature by integrating the organism-environment interaction model, the protection motivation theory, and health belief model, to investigate the mechanisms by which “untact” buying behavior is increased under COVID-19 outbreak. That is, individuals’ consumption behavior can be function as a protective behavior (e.g., “untact” buying behavior) may be partly fueled by their intrinsic personality traits like intolerance of uncertainty in epidemic outbreak situations. Besides, according to the health belief model, perceived risk of COVID-19 variants and protection motivation can work together and act as the underlying mechanism that accelerates the effects of individuals’ intolerance of uncertainty on “untact” buying behavior. Practically, our research offers important implications for e-commerce managers and companies to maintain economic growth during the epidemic. The dynamic changes of COVID-19 in the whole world may provide clues to how e-commerce managers and companies may preemptively respond to changing market demands. Specifically, consumers’ perceived risk of COVID-19 may be improved by their intolerance of uncertainty, which in turn leads to more online shopping and less offline shopping through enhancing their protection motivation. In addition, individuals’ protection motivation to reduce the risk of COVID-19 infection need e-commerce companies and retailers do a good job of disinfection in the transaction process during COVID-19 outbreak period.

## Limitations and Directions for Future Research

Several limitations should take into consideration when we interpret the findings of the current study. Firstly, this study only explored the impact of intolerance of uncertainty on “untact” buying behavior. Previous study indicates that other individual differences such as need for cognition (Cacioppo and Petty, 1982) and cognitive consumer innovativeness (Vandecasteele and Geuens, 2008) may also promote online buying behavior (Aquino et al., 2018). Future studies may examine the roles of need for cognition and cognitive consumer innovativeness in understanding individuals’ “untact” buying behavior. Secondly, this study only focused on purchasing in general without referring to a specific product. Future investigation may focus on a specific product such as food or clothing. Thirdly, this study adopted a cross-sectional design, the causal inferences need to be avoided. Besides, the indirect effects of intolerance of uncertainty on “untact” buying behavior through perceived risk of COVID-19 variants and the path from perceived risk of COVID-19 variants to protection motivation were significant but low. Future studies may consider using longitudinal or experimental designs to verify the robustness of this chain mediation model. Fourthly, all variables were assessed via self-report measures, which might weaken the validity of the present study. At last, since the current investigation was conducted in China characterized by collectivist culture, it is necessary to be cautious to infer conclusions to other cultural backgrounds.

## Conclusion

The present study found that participants reporting more “untact” buying behavior usually have higher intolerance of uncertainty than their counterparts. Furthermore, perceived risk of COVID-19 variants and protection motivation acted as partial mediators of the relationship between intolerance of uncertainty and “untact” buying behavior independently. More interestingly, intolerance of uncertainty can also link to “untact” buying behavior through the path of the perceived risk of COVID-19 variants-protection motivation. The results demonstrated that the perceived risk of COVID-19 variants acts as a bridge between intolerance of uncertainty and “untact” buying behavior. Due to the concealment of the infectious diseases and the instability of the epidemic control effect, providing “untact” service based on advanced information technology is very necessary for the post-epidemic period. By providing “untact” buying services, individuals can protect themselves from COVID-19 via satisfying safety needs. It may also help individuals maintain mental health by alleviating negative emotional experiences like anxiety and worry during the COVID-19 period.

## Data Availability Statement

The raw data supporting the conclusions of this article will be made available by the authors, without undue reservation.

## Author Contributions

SZ constructed the model, carried out the study, performed the statistical analysis, and drafted the manuscript. BY provided the idea, participated in the interpretation of the data, and revised the manuscript. WW participated in the interpretation of the data and revised the manuscript. YZ participated in its design and the interpretation of the data. All authors contributed to the article and approved the submitted version.

## Conflict of Interest

The authors declare that the research was conducted in the absence of any commercial or financial relationships that could be construed as a potential conflict of interest.

## Publisher’s Note

All claims expressed in this article are solely those of the authors and do not necessarily represent those of their affiliated organizations, or those of the publisher, the editors and the reviewers. Any product that may be evaluated in this article, or claim that may be made by its manufacturer, is not guaranteed or endorsed by the publisher.

## References

[B1] Akter S. Ashrafi T. Waligo V. (2021). Changes in consumer purchasing behavior due to COVID-19 pandemic. *J. Mark. Consumer Res.* 2021 33–46.

[B2] Ali B. (2020). Impact of COVID-19 on consumer buying behavior toward onlineshopping in Iraq. *Econ. Stud. J.* 18 267–280.

[B3] Al-Rasheed M. (2020). Protective behavior against COVID-19 among the public in kuwait: an examination of the protection motivation theory, trust in government and sociodemographic factors. *Soc. Work Public Health* 35 546–556. 10.1080/19371918.2020.1806171 32970542

[B4] Aquino A. Picconi L. Alparone F. R. (2018). Validation of the italian version of the need for cognition scale-short version. *BPA Appl. Psychol. Bull.* 66 18–29. 10.26387/bpa.283.2

[B5] Asmundson G. J. Taylor S. (2020). Coronaphobia: fear and the 2019-nCoV outbreak. *J. Anxiety Dis.* 70:102196. 10.1016/j.janxdis.2020.102196 32078967 PMC7134790

[B6] Bae S. Y. Chang P.-J. (2020). The effect of coronavirus disease-19 (COVID-19) risk perception on behavioural intention towards ‘untact’ tourism in south korea during the first wave of the pandemic (March 2020). *Curr. Issues Tourism* 24 1017–1035. 10.1080/13683500.2020.1798895

[B7] Bakioğlu F. Korkmaz O. Ercan H. (2020). Fear of COVID-19 and positivity: mediating role of intolerance of uncertainty, depression, anxiety, and stress. *Int. J. Mental Health Addict.* [Epub ahead of print], 10.1007/s11469-020-00331-y 32837421 PMC7255700

[B8] Bala S. (2021). *China’s Gen Z Consumers Drive Growth of Domestic Brands as Spending Power Rises.* CNBC. Available online at: https://www.cnbc.com/2021/02/17/china-consumer-trends-gen-z-drive-growth-of-domestic-brands.html (accessed December 28, 2021).

[B9] Bali S. Stewart K. A. Pate M. A. (2016). Long shadow of fear in an epidemic: fearonomic effects of ebola on the private sector in nigeria. *BMJ Global Health* 1:111. 10.1136/bmjgh-2016-000111 28588965 PMC5321397

[B10] Bish A. Yardley L. Nicoll A. Michie S. (2011). Factors associated with uptake of vaccination against pandemic influenza: a systematic review. *Vaccine* 29 6472–6484. 10.1016/j.vaccine.2011.06.107 21756960

[B11] Brouwers M. C. Sorrentino R. M. (1993). Uncertainty orientation and protection motivation theory: the role of individual differences in health compliance. *J. Personal. Soc. Psychol.* 65 102–112. 10.1037/0022-3514.65.1.102

[B12] Brug J. Aro A. R. Oenema A. Zwart O. D. Richardus J. H. Bishop G. D. (2004). SARS risk perception, knowledge, precautions, and information sources, the netherlands. *Emerg. Infect. Dis.* 10 1486–1489. 10.3201/eid1008.040283 15496256 PMC3320399

[B13] Buhr K. Dugas M. J. (2002). The intolerance of uncertainty scale: psychometric properties of the english version. *Behav. Res. Ther.* 40 931–945. 10.1016/S0005-7967(01)00092-4 12186356

[B14] Cacioppo J. T. Petty R. E. (1982). The need for cognition. *J. Personal. Soc. Psychol.* 42 116–131. 10.1037/0022-3514.42.1.116

[B15] Carleton R. N. (2016). Into the unknown: a review and synthesis of contemporary models involving uncertainty. *J. Anxiety Dis.* 39 30–43. 10.1016/j.janxdis.2016.02.007 26945765

[B16] Carleton R. N. Norton M. A. P. J. Asmundson G. J. G. (2007). Fearing the unknown: a short version of the intolerance of uncertainty scale. *J. Anxiety Dis.* 21 105–117. 10.1016/j.janxdis.2006.03.014 16647833

[B17] Champion V. L. Skinner C. S. (2008). The health belief model. *Health Behav. Health Educ. Theory Res. Pract.* 4 45–65.

[B18] Cowling B. J. Ng D. M. Ip D. K. Liao Q. Lam W. W. Wu J. T. (2010). Community psychological and behavioral responses through the first wave of the 2009 influenza A (H1N1) pandemic in hong kong. *J. Infect. Dis.* 202 867–876. 10.1086/655811 20677945

[B19] Curran P. J. West S. G. Finch J. F. (1996). The robustness of test statistics to non-normality and specification error in confirmatory factor analysis. *Psychol. Methods* 1 16–29. 10.1037/1082-989x.1.1.16

[B20] Dai B. Fu D. Meng G. Li Q. Liu X. Liu B. (2020). The effects of governmental and individual predictors on COVID-19 protective behaviors in China: a path analysis model. *Public Administr. Rev.* 80 797–804. 10.1111/puar.13236 32836438 PMC7276878

[B21] Di Giuseppe K. Taylor A. J. (2021). Investigating how intolerance of uncertainty and emotion regulation predict experiential avoidance in non-clinical participants. *Psychol. Stud.* 66 1–10. 10.1007/s12646-021-00602-1

[B22] Dugas M. J. Hedayati M. Karavidas A. Buhr K. Francis K. Phillips N. A. (2005). Intolerance of uncertainty and information processing: evidence of biased recall and interpretations. *Cogn. Ther. Res.* 29 57–70. 10.1007/s10608-005-1648-9

[B23] Durak Batıgün A. Şenkal Ertürk Ý (2021). COVID-19 associated psychological symptoms in turkish population: a path model. *Curr. Psychol.* [Epub ahead of print], 10.1007/s12144-021-02026-6 34230790 PMC8247107

[B24] Fitzsimmons J. A. Fitzsimmons M. J. Bordoloi S. (2008). *Service Management: Operations, Strategy, and Information Technology.* New York: McGraw-Hill.

[B25] Floyd D. L. Prentice-Dunn S. Rogers R. W. (2000). A meta-analysis of research on protection motivation theory. *J. Appl. Soc. Psychol.* 30 407–429. 10.1111/j.1559-1816.2000.tb02323.x

[B26] Freeston M. H. Rhéaume J. Letarte H. Dugas M. J. Ladouceur R. (1994). Why do people worry? *Personal. Indiv. Diff.* 17 791–802. 10.1016/0191-8869(94)90048-5

[B27] Golets A. Farias J. Pilati R. Costa H. (2021). COVID-19 pandemic and tourism: the impact of health risk perception and intolerance of uncertainty on travel intentions. *Curr. Psychol.* [Epub ahead of print]. 10.20944/preprints202010.0432.v1 34539156 PMC8436199

[B28] Greco V. Roger D. (2001). Coping with uncertainty: the construction and validation of a new measure. *Personal. Indiv. Diff.* 31 519–534. 10.1016/S0191-8869(00)00156-2

[B29] Hayes A. F. (2013). *Introduction to Mediation, Moderation, and Conditional Process Analysis: A Regression-Based Approach.* New York: The Guilford Press, 10.1111/jedm.12050

[B30] Hayes A. F. (2017). *Introduction to Mediation, Moderation, and Conditional Process Analysis:A Regression-Based Approach.* New York: Guilford Publications.

[B31] Hillen M. A. Gutheil C. M. Strout T. D. Smets E. M. Han P. K. (2017). Tolerance of uncertainty: conceptual analysis, integrative model, and implications for healthcare. *Soc. Sci. Med.* 180 62–75. 10.1016/j.socscimed.2017.03.024 28324792

[B32] Ibuka Y. Chapman G. B. Meyers L. A. Li M. Galvani A. P. (2010). The dynamics of risk perceptions and precautionary behavior in response to 2009 (H1N1) pandemic influenza. *BMC Infect. Dis.* 10:1–11. 10.1186/1471-2334-10-296 20946662 PMC2964717

[B33] Jmour A. (2020). “«Man-machine» interaction: the determinants of the untact service’s use,” in *Proceeding of the International Conference on Digital Economy*, 105–114. 10.1007/978-3-030-64642-4_9

[B34] Jose R. Narendran M. Bindu A. Beevi N. Manju L. Benny P. V. (2021). Public perception and preparedness for the pandemic COVID 19: a health belief model approach. *Clin. Epidemiol. Global Health* 9 41–46. 10.1016/j.cegh.2020.06.009 33521389 PMC7837111

[B35] Kagan J. (1972). Motives and development. *J. Personal. Soc. Psychol.* 22 51–66. 10.1037/h0032356 5013358

[B36] Kaspar K. (2020). Motivations for social distancing and app use as complementary measures to combat the COVID-19 pandemic: quantitative survey study. *J. Med. Int. Res.* 22:21613. 10.2196/21613 32759100 PMC7458661

[B37] Keren G. Gerritsen L. E. (1999). On the robustness and possible accounts of ambiguity aversion. *Acta Psychol.* 103 149–172. 10.1016/S0001-6918(99)00034-7

[B38] Khan H. (2021). Examining the buying behavior of online consumers in india during the pandemic COVID-19. *J. Manage. Train. Industr.* 8 1–23. 10.12792/JMTI.8.2.1

[B39] Kim A. Sung Y. (2020). My privacy and control matter: understanding motivations for using untact services. *Cyberpsychol. Behav. Soc. Networking* 24 426–431. 10.1089/cyber.2020.0350 33337264

[B40] Koerner N. Dugas M. J. (2008). An investigation of appraisals in individuals vulnerable to excessive worry: the role of intolerance of uncertainty. *Cogn. Ther. Res.* 32 619–638. 10.1007/s10608-007-9125-2

[B41] Laugesen N. Dugas M. J. Bukowski W. M. (2003). Understanding adolescent worry: the application of a cognitive model. *J. Abnormal Child Psychol.* 31 55–64. 10.1023/A:1021721332181 12597699

[B42] Lee S. M. Lee D. (2020). “Untact”: a new customer service strategy in the digital age. *Service Bus.* 14 1–22. 10.1007/s11628-019-00408-2

[B43] Lee S. M. Lee D. Kim Y. S. (2019). The quality management ecosystem for predictive maintenance in the industry 4.0 era. *Int. J. Q. Innovat.* 5 1–11. 10.1109/TII.2019.2915846

[B44] Lerner R. M. Lerner J. V. Almerigi J. Theokas C. (2006). “Dynamics of individual context relations in human development: a developmental systems perspective,” in *Comprehensive Handbook of Personality and Psychopathology*, eds Thomas J. C. Segal D. L. Hersen M. Thomas J. C. Segal D. L. Hersen M. (John Wiley & Sons).

[B45] Li J. Hallsworth A. G. Coca-Stefaniak J. A. (2020). Changing grocery shopping behaviours among Chinese consumers at the outset of the COVID-19 outbreak. *Tijdschrift Voor Economische En Sociale Geografie* 111 574–583. 10.1111/tesg.12420 32836486 PMC7307130

[B46] Li Z. Ma Z. (2018). Factors influencing online shopping intention and their complex relationships:based on PLS-SEM and BN. *J. Stat. Inform.* 33 110–116.

[B47] Liu C. (2020). *Top 10 Ecommerce Retailers Will Grow Their Market Share to 60.1% in 2020.* Insider Intelligence. Available online at: https://www.emarketer.com/content/top-10-ecommerce-retailers-will-grow-their-share-60-2020 (accessed July 14, 2020)

[B48] Lwin M. O. Stanaland A. J. Chan D. (2010). Using protection motivation theory to predict condom usage and assess HIV health communication efficacy in singapore. *Health Commun.* 25 69–79. 10.1080/10410230903473540 20390672

[B49] Mertens G. Gerritsen L. Duijndam S. Salemink E. Engelhard I. M. (2020). Fear of the coronavirus (COVID-19): predictors in an online study conducted in march 2020. *J. Anxiety Dis.* 74:102258. 10.1016/j.janxdis.2020.102258 32569905 PMC7286280

[B50] Milne S. Sheeran P. Orbell S. (2000). Prediction and intervention in health-related behavior: a meta-analytic review of protection motivation theory. *J. Appl. Soc. Psychol.* 30 106–143. 10.1111/j.1559-1816.2000.tb02308.x

[B51] Perić G. Dramiæanin S. Coniæ M. (2021). The impact of serbian tourists’ risk perception on their travel intentions during the COVID-19 pandemic. *Eur. J. Tourism Res.* 27 2705–2705.

[B52] Posey C. Roberts T. L. Lowry P. B. (2015). The impact of organizational commitment on insiders’ motivation to protect organizational information assets. *J. Manage. Inform. Syst.* 32 179–214. 10.1080/07421222.2015.1138374

[B53] Rogers R. (1983). *Cognitive and Physiological Processes in Fear Appeals and Attitude Change: A Revised Theory of Protection Motivation.* New York: Guilford Press.

[B54] Rogers R. W. (1975). A protection motivation theory of fear appeals and attitude change1. *J. Psychol.* 91 93–114. 10.1080/00223980.1975.9915803 28136248

[B55] Rosen N. O. Knäuper B. (2009). A little uncertainty goes a long way: state and trait differences in uncertainty interact to increase information seeking but also increase worry. *Health Commun.* 24 228–238. 10.1080/10410230902804125 19415555

[B56] Rosen N. O. Ivanova E. Knäuper B. (2014). Differentiating intolerance of uncertainty from three related but distinct constructs. *Anxiety Stress Coping* 27 55–73. 10.1080/10615806.2013.815743 23849047

[B57] Rosenstock I. M. (1974). Historical origins of the health belief model. *Health Educ. Monogr.* 2 328–335. 10.1177/10901981740020040 299611

[B58] Satici B. Saricali M. Satici S. A. Griffiths M. D. (2020). Intolerance of uncertainty and mental wellbeing: serial mediation by rumination and fear of COVID-19. *Int. J. Mental Health and Add.* [Epub ahead of print], 10.1007/s11469-020-00305-0 32427165 PMC7228430

[B59] Schmees R. (2020). *The Role of Cognitive Appraisals and Past Protective Behavior in Future Protection Motivation: Applying Protection Motivation Theory to the COVID-19 Pandemic Ph. D, Thesis.* University of Twente.

[B60] Shahnazi H. Ahmadi-Livani M. Pahlavanuadeh B. Rajabi A. Hamrah M. S. Charkazi A. (2020). Assessing preventive health behaviors from COVID-19 based on the health belief model (HBM) among people in golestan province: a cross-sectional study in northern Iran. *Infect. Dis. Prevent. Med.* 9:157. 10.21203/rs.3.rs-24871/v1 PMC7671178 33203453

[B61] Sharifirad G. Yarmohammadi P. Sharifabad M. Rahaei Z. (2014). Determination of preventive behaviors for pandemic influenza A/H1N1 based on protection motivation theory among female high school students in Isfahan. Iran. *J. Educ. Health Promot.* 3 36–41. 10.4103/2277-9531.127556 24741647 PMC3977398

[B62] Sjöberg L. (2000). Factors in risk perception. *Risk Analysis Off. Publi. Soc. Risk Analy.* 20 1–11. 10.1111/0272-4332.00001 10795334

[B63] Slovic P. (1987). Perception of risk. *Science* 236 280–285. 10.1126/science.3563507 3563507

[B64] Statista Research Department (2021). *Online Shopping Transaction Volume in South Korea from 2009 to 2020.* Statista Research Department. Available online at: https://www.statista.com/statistics/280922/b2c-e-commerce-sales-in-south-korea/ (accessed April 30, 2021).

[B65] Taha S. Matheson K. Cronin T. Anisman H. (2014). Intolerance of uncertainty, appraisals, coping, and anxiety: the case of the 2009 H 1 N 1 pandemic. *Br. J. Health Psychol.* 19 592–605. 10.1111/bjhp.12058 23834735

[B66] Tooher R. Collins J. E. Street J. M. Braunack-Mayer A. Marshalla H. (2013). Community knowledge, behaviors and attitudes about the 2009 H1N1 influenza pandemic: a systematic review. *Influenza Other Respiratory Viruses* 7 1316–1327. 10.1111/irv.12103 23560537 PMC4634241

[B67] Tull M. T. Barbano A. C. Scamaldo K. M. Richmond J. R. Edmonds K. A. Rose J. P. (2020). The prospective influence of COVID-19 affective risk assessments and intolerance of uncertainty on later dimensions of health anxiety. *J. Anxiety Dis.* 75:102290. 10.1016/j.janxdis.2020.102290 32823216 PMC7422821

[B68] van der Weerd W. Timmermans D. R. Beaujean D. J. Oudhoff J. van Steenbergen J. (2011). Monitoring the level of government trust, risk perception and the intention of the general public to adopt protective measures during the influenza a (H1N1) pandemic in the Netherlands. *BMC Public Health* 11:575. 10.1186/1471-2458-11-575 21771296 PMC3152536

[B69] Vandecasteele B. Geuens M. (2008). Motivated consumer innovativeness: concept and measurement. *Int. J. Res. Mark.* 8 1–9. 10.1016/j.ijresmar.2010.08.004

[B70] Wu L. J. Wang J. N. Qi X. D. (2016). Validity and reliability of the intolerance of uncertainty scale-12 in middle school students. *Chin. Mental Health J.* 30 700–706.

[B71] Yastibaş C. Akpinar Z. (2021). Personality traits and health-related quality of life in irritable bowel syndrome (IBS) patients: the mediating role of illness perceptions. *Psychol. Stud.* 66:200. 10.1007/s12646-021-00618-7

[B72] Yıldırım M. Güler A. (2020). Factor analysis of the COVID-19 perceived risk scale: a preliminary study. *Death Stud.* [Epub ahead of print], 10.1080/07481187.2020.1784311 32584201

[B73] Zheng D. Luo Q. Ritchie B. W. (2021). Afraid to travel after COVID-19? self-protection, coping and resilience against pandemic ‘travel fear’. *Tourism Manage.* 83:104261. 10.1016/j.tourman.2020.104261

